# Asymmetric Hysteresis Loops in Structured Ferromagnetic Nanoparticles with Hard/Soft Areas †

**DOI:** 10.3390/nano11030800

**Published:** 2021-03-21

**Authors:** Joscha Detzmeier, Kevin Königer, Tomasz Blachowicz, Andrea Ehrmann

**Affiliations:** 1 Faculty of Engineering and Mathematics, Bielefeld University of Applied Sciences, 33619 Bielefeld, Germany; joscha.detzmeier@fh-bielefeld.de (J.D.); kevin.koeniger@fh-bielefeld.de (K.K.); 2 Center for Science and Education, Institute of Physics, Silesian University of Technology, 44-100 Gliwice, Poland; tomasz.blachowicz@polsl.pl

**Keywords:** pseudo-exchange bias, minor loop, micromagnetic simulation, OOMMF, spintronics

## Abstract

Horizontally shifted and asymmetric hysteresis loops are often associated with exchange-biased samples, consisting of a ferromagnet exchange coupled with an antiferromagnet. In purely ferromagnetic samples, such effects can occur due to undetected minor loops or thermal effects. Simulations of ferromagnetic nanostructures at zero temperature with sufficiently large saturation fields should not lead to such asymmetries. Here we report on micromagnetic simulations at zero temperature, performed on sputtered nanoparticles with different structures. The small deviations of the systems due to random anisotropy orientations in the different grains can not only result in strong deviations of magnetization reversal processes and hysteresis loops, but also lead to distinctly asymmetric, horizontally shifted hysteresis loops in purely ferromagnetic nanoparticles.

## 1. Introduction

The exchange bias (EB) effect describes a phenomenon that occurs in ferromagnet/antiferromagnet systems due to an exchange coupling at the interface and leads to a shift of the hysteresis loop, often in combination with an asymmetry of the loop [1]. After firstly being found in Co/CoO core/shell nanoparticles [2], the exchange bias is now mostly investigated in thin-film systems [3,4,5,6]. Technologically, the effect is particularly relevant for hard disk read heads, spin valves and other spintronic devices [7,8,9,10].

Although the origin of the EB is not yet fully understood quantitatively, there is general agreement that the interface between a ferromagnet and an antiferromagnet—or ferrimagnet—plays a crucial role in this effect [11,12,13]. Recently, the additional influence of long-range interactions in the antiferromagnet has been shown [14,15]. On the other hand, this means that in purely ferromagnetic systems, regardless of size or shape, neither a shift of the hysteresis loop nor an asymmetry of the hysteresis loop is to be expected.

Nevertheless, only few studies report on such effects. In particular, magnetization measurements using the magneto-optical Kerr effect (MOKE), where only magnetization differences are detectable while the absolute magnetization cannot be measured, do not show a possible vertical shift of the hysteresis loop, so that this method is prone to erroneously measuring minor loops that appear to be completely closed, while saturation is not yet reached [16]. On the other hand, exchange bias-like loop shifts were reported in pure antiferromagnets, where they were attributed to uncompensated spins inside the antiferromagnet, which led to field-resistant magnetization [17].

Here we show micromagnetic simulations using the Object Oriented MicroMagnetic Framework (OOMMF) at zero temperature, performed on symmetrical sputtered nanoparticles with different shapes and holes or slits inside. Such structures are interesting, since they can often be used to prepare quaternary memory devices in which two (or even more) bits can be stored in one storage position [18]. Our results show that not only the small deviations of the investigated systems due to random anisotropy orientations in the different grains can lead to strong deviations of magnetization reversal processes and hysteresis loops, but also that distinctly asymmetric, horizontally shifted hysteresis loops can occur in a purely ferromagnetic nanoparticle.

## 2. Materials and Methods

**Figure 1:**
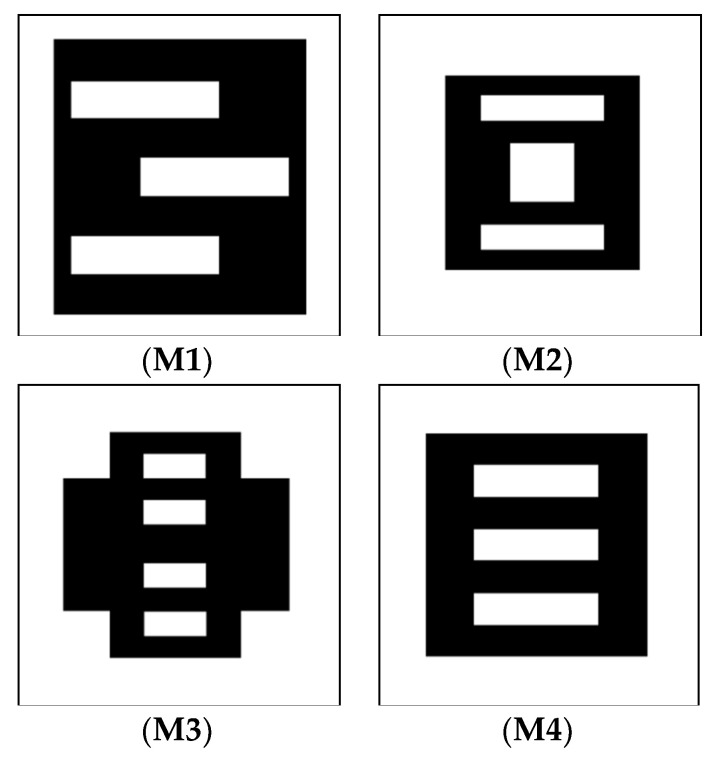
Different masks M1-M4 with slots under investigation. Black areas depict Fe, while white areas are empty—filled with air.

Different nanostructures were modeled, as depicted in Figure 1. The lateral dimensions of the frames are always 100 nm × 100 nm, and the height was defined as 10 nm.

Based on the results of these simulations and further preliminary tests, additional structures were developed with lateral dimensions of 500 nm × 500 nm (465 nm/600 nm) in case of square (hexagonal / irregular) patterns and a thickness of 50 nm. Parts of the samples have a reduced thickness of 25 nm (visible by gray areas in the respective snapshots of the magnetization reversal processes), while black and white show iron of full height and air again, respectively.

For the simulations, OOMMF was used [19], which is based on finite differences for the meshing and dynamic solution of the Landau-Lifshitz-Gilbert (LLG) equation of motion [20]. The material parameters were chosen as typical literature values for iron (Fe): magnetization at saturation M_S_ = 1700·10^3^ A/m, exchange constant A = 21·10^−12^ J/m, magneto-crystalline anisotropy constant K_1_ = 48·10^3^ J/m^3^.

Since such nanostructures are usually fabricated by electron beam lithography [21], the anisotropy axes in neighboring cubic cells with 5 nm^3^ were randomly selected so that the configurational anisotropy dominantly determines the magnetization reversal [22]. Setting the Gilbert damping constant at α = 0.5 results in simulations of a realistic quasi-static case. External magnetic fields were applied in the sample plane and were swept between different maximum fields at diverse angles. It must be mentioned that the temperature was set to 0 K to avoid thermal fluctuations which could hinder the magnetization reversal process.

Parts of the results were published in [23].

## 3. Results and Discussion

**Figure 2:**
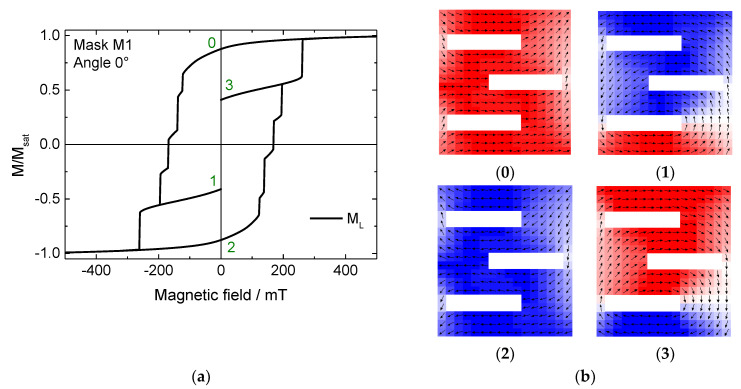
(**a**) Hysteresis loop and (**b**) snapshots of stable intermediate states #0–3, simulated for mask M1 under a field angle of 0°.

The original goal of this study was to investigate possible structures for quaternary storage applications. Figure 2 shows this effect exemplarily for mask M1 under an angle of 0° (horizontal orientation in Figure 1). Figure 2a depicts the longitudinal magnetization component M_L_, parallel to the external magnetic field. Here, the field sweep was stopped only for the widest steps, decreasing the external magnetic field to 0 in order to investigate the stability of the different states achieved in this way at remanence (states 1 and 3 in the figure). Thus, in addition to the common two states, two further stable states at remanence could be verified. The snapshots corresponding to the numbers of the intermediate states in Figure 2a are depicted in Figure 2b.

Here it becomes clear why this nanoparticle structure is well suited to provide more than one stable state at remanence: the different areas of the structure do not switch the magnetization simultaneously, and these partially switched states correspond to the steps along the slope of the hysteresis loop (Figure 2a). 

However, it must be mentioned that the other steps are either not correlated with stable intermediate states (not shown here) or are too narrow (in the sense of the step width) to be technologically important, and therefore have not been further investigated here. Nevertheless, the different slit positions in the sample allow interesting magnetic states and should therefore be examined more in detail in the near future by varying all dimensions of this nano-object.

**Figure 3:**
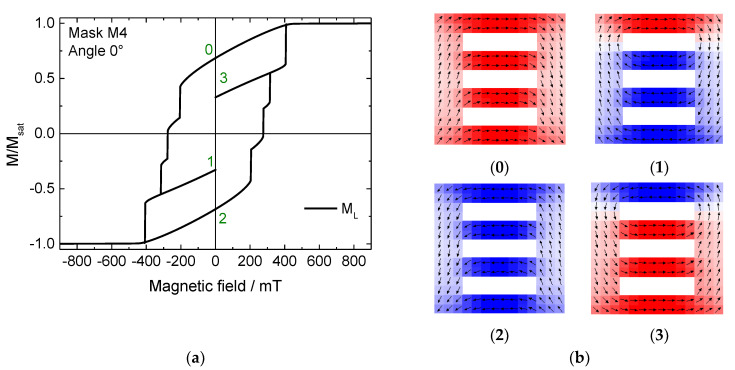
(**a**) Hysteresis loop and (**b**) snapshots of stable intermediate states #0–3, simulated for mask M4 under a field angle of 0°.

A very similar effect is found in mask M4, as shown in Figure 3. Here, each bar switches successively, which theoretically enables even eight different states at remanence. The narrower steps, however, were again not tested because they are practically less relevant due to their restricted width.

**Figure 4:**
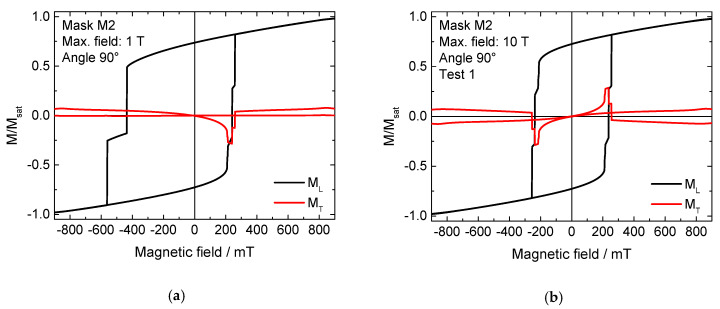

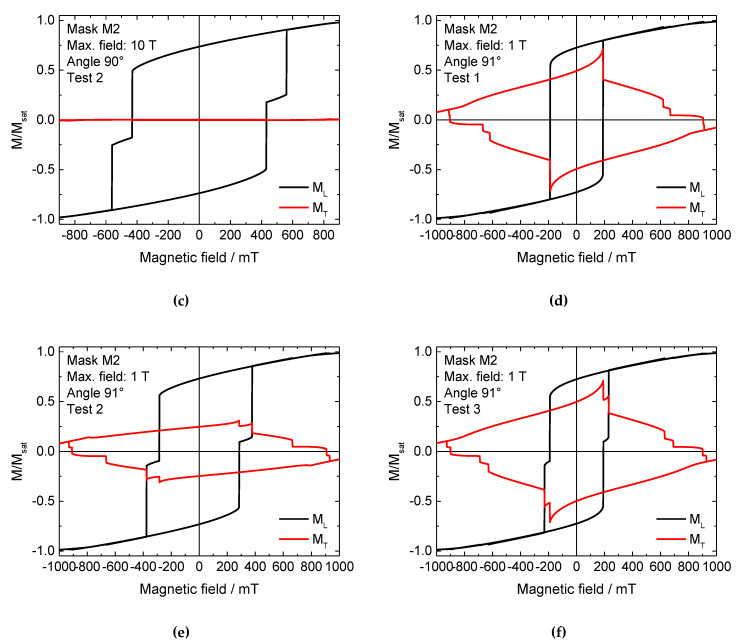
(**a**–**f**) Longitudinal and transverse hysteresis loops ML and MT, simulated for mask M2 under a field angle of ~90° applying different maximum fields, incl. different runs with a maximum external magnetic field of 10 T.

Similar effects were found for sample M2 at an angle of 0°. However, the rotation of the external magnetic field to an orientation of 90° led to an unexpected finding. As Figure 4a shows, in this case a horizontally shifted, asymmetric hysteresis curve occurs, which shows the typical form of, e.g., Fe/MnF_2_ exchange-biased thin-film systems [24]. As discussed above, in the case of a pure ferromagnet such a finding should be attributed to the measurement of a minor loop.

While the maximum fields of ± 1 T applied here are already quite large, it is known that saturation fields can be much larger than coercive fields; therefore, a maximum field of 10 T was applied in the next simulations (field range not fully shown in Figure 4). This order of magnitude is accessible with common magnets in cryostats. Two of the results are depicted in Figure 4b,c. It should be mentioned that all test parameters were kept identical, the only variable being the angle of the magneto-crystalline anisotropy per grain, which is arbitrarily chosen with each new simulation run, corresponding to the situation of sputtered samples in reality. Both experiments yielded symmetrical curves. Nevertheless, in spite of simulating the same situation, different coercive fields and also different magnetization reversal processes were found, as the strongly different transverse hysteresis loops M_T_ show. Comparing Figure 4a with these 10 T-saturated measurements, it is obvious that the asymmetric loop in Figure 4a contains results of both hysteresis loops shown in Figure 4b,c, underlining the idea that Figure 4a shows a pseudo-EB due to simulating a minor loop.

Next, it was tested whether a slight symmetry breaking by a further rotation of the sample by 1° would increase the reproducibility of the results. This approach works well in masks M1 and M4, where a rotation of the external magnetic field can be used to define whether magnetization reversal starts in the “top” or the “bottom” horizontal bar. However, as depicted in Figure 4c–f, this approach was not successful. All longitudinal as well as transverse magnetization components differ clearly. It is also visible that the transverse magnetization components are saturated only at absolute fields greater than 900 mT, whereas the longitudinal loops seem to be saturated at less than half of these fields. This underlines the earlier finding that minor loops can remain undiscovered in experiments where the transverse magnetization components are often not measured separately [16], and again shows the lack of reproducibility of magnetization reversal processes in nanoparticles with small regions that can remain unchanged. Very similar results were found in the sample M3 at an angle of 0° (not shown here), where three different magnetization reversal processes were also observed in subsequent simulations.

While the lack of reliability in some of the samples is problematic for a possible technological application, the pseudo-exchange bias is even technologically relevant for spintronic applications. It has to be emphasized that although this effect shows a similar behavior to exchange bias, it is not due to exchange coupling between different magnetic materials or between different species within one magnetic material [3] and therefore cannot be considered as exchange bias, but is based on minor loops. Such minor loops are usually avoided in most measurements, since they can lead to misinterpretations. However, the possibility to prepare a purely ferromagnetic system with a pseudo-EB could offer new possibilities to create simpler spin-valves and other spintronics elements that normally require many layers, including a ferromagnetic layer pinned by an antiferromagnet to increase its switching field. If it is possible to prepare layers with an intrinsic pseudo-EB due to a sophisticated layer geometry, resulting in neighboring areas with soft and with hard magnetic behavior, the antiferromagnet can be omitted, and thus spintronics devices can be produced from simpler layer stacks.

**Figure 5:**
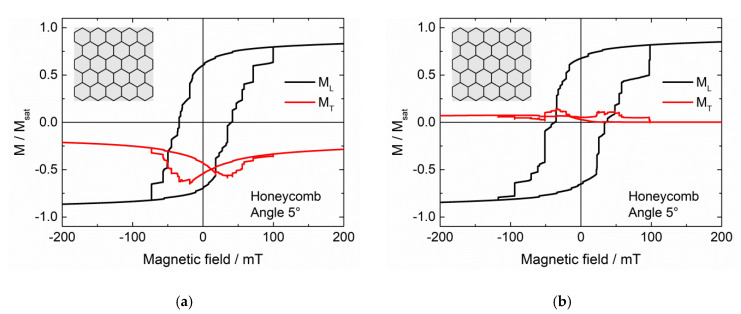
(**a**) Longitudinal and transverse hysteresis loops M_L_ and M_T_, simulated for a hexagonal tessellation (cf. insets), (**a**) after setting a large magnetic field of 5 T; (**b**) starting from zero field.

This is why in the next part of this study, larger samples with repeated unit cells were simulated. By continuous repetitions of these simulations, we found that a higher thickness (now 50 nm instead of 10 nm in the first part of the study) and a tessellation pattern instead of a single structured nanoparticle lead to more reproducible results, as compared to those shown before. It must be mentioned that the starting configuration nevertheless influences the results, as visible by a comparison with Figure 5a,b. For systems with very large saturation fields, i.e., with the possibility to mimic an exchange bias by a minor loop, this initial saturation can be assumed to work in the same way as the cooling field in classical EB systems.

**Figure 6:**
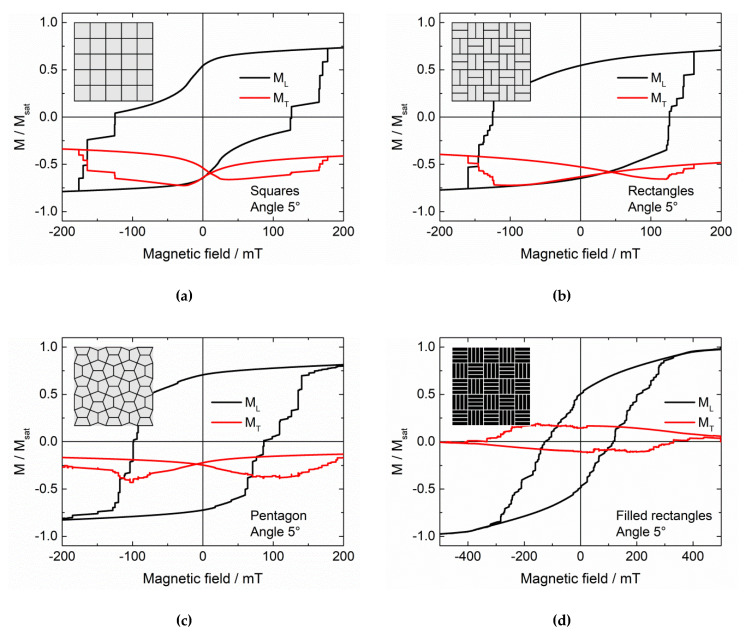
(**a**–**d**) Longitudinal and transverse hysteresis loops ML and MT, simulated for different tessellations (cf. insets) after setting a large magnetic field of 5 T.

Figure 6 thus shows longitudinal and transverse hysteresis loops, simulated after setting a large external magnetic field of + 5 T to reach positive saturation, for different tessellations, as depicted in the insets.

In most cases (Figure 6a–c), it is possible to create asymmetric hysteresis loops by the initial field setting. This does not work, however, in the case of Figure 6d, where most of the nanoparticle has the maximum height, separated by thin lines of air. These first results suggest that a higher “mesh” connected with thinner filled magnetic areas is necessary to reach this effect.

It must also be mentioned that this asymmetry is usually observed in the transverse rather than in the longitudinal hysteresis loop. Besides, for the repeated simulations we always found vertical shifts of the transverse magnetization to negative values, while the shape of the longitudinal hysteresis varied slightly due to the arbitrary orientation of the magneto-crystalline anisotropy axes in the single grains. This means that for a potential application, the transverse magnetization component—or the magnetization in another direction—may be more suitable than the longitudinal one.

Comparing these structures with the first set M1–M4, it must also be mentioned that the reliability of magnetization reversal in terms of coercive fields and the shape of the longitudinal hysteresis loop is given in the tessellation structures, if the samples are examined from a macroscopic point of view, i.e., according to their overall magnetization. However, from a microscopic point of view, the magnetization reversal processes on small scales may still differ due to the variations of the angle of the magneto-crystalline anisotropy in each single cell. Thus the order or reversal of the single tiles of these tessellations will differ from one simulation run to the next one.

Generally, it must be emphasized that the effect depicted here for some examples is not an equivalent to an exchange bias system, which usually consists of a ferromagnet exchange-coupled to an antiferromagnet, but to a combination of a harder and a softer ferromagnet. Opposite to spin valves and similar spintronics devices, the system does not contain layers of hard and soft ferromagnets, but both properties are intermixed inside a single ferromagnetic nanostructure. Typically, the hard ferromagnetic parts are the thin, higher lines with their strong shape anisotropy, while the larger, flatter parts in between are responsible for the soft ferromagnetic behavior.

To enable a reliable utilization of this effect, it is necessary to test its stability against small deviations of the structure and the angle under which the external magnetic field is applied, which will be done in the next study.

## 4. Conclusions

Pure ferromagnetic nanoparticles with internal slits or with lines of increased height were investigated by micromagnetic simulations. In several cases, clearly asymmetric hysteresis loops were found, which is normally correlated with exchange bias systems, but can here be attributed to simulating minor loops. The asymmetry was especially well visible in the transverse hysteresis loops.

While the asymmetry in an exchange bias system stems from the exchange-coupling between ferro- and antiferromagnet, here it can be attributed to the interaction between a hard and an easy ferromagnet which are, unlike spin-valves and other common thin-film systems, not spatially separated, but intermixed.

Future research will show whether such structures can reliably be used instead of exchange bias systems in different spintronics devices.

## Data Availability

All data gained during the study are shown in this paper.
